# Unraveling protein dynamics to understand the brain – the next molecular frontier

**DOI:** 10.1186/s13024-022-00546-8

**Published:** 2022-06-18

**Authors:** Kyle D. Brewer, Sophia M. Shi, Tony Wyss-Coray

**Affiliations:** 1 ChEM-H, Stanford University, Stanford, CA USA; 2 Department of Neurology and Neurological Sciences, Stanford University School of Medicine, Stanford, CA USA; 3 Wu Tsai Neurosciences Institute, Stanford University, Stanford, CA USA; 4 Department of Chemistry, Stanford University, Stanford, CA USA; 5 Phil and Penny Knight Initiative for Brain Resilience, Stanford University, Stanford, CA USA

**Keywords:** Protein labeling, Protein tracking, Plasma, Blood–brain barrier, Bioorthogonal labeling, Proteomics, Mass spectrometry

## Abstract

The technological revolution to measure global gene expression at the single-cell level is currently transforming our knowledge of the brain and neurological diseases, leading from a basic understanding of genetic regulators and risk factors to one of more complex gene interactions and biological pathways. Looking ahead, our next challenge will be the reliable measurement and understanding of proteins. We describe in this review how to apply new, powerful methods of protein labeling, tracking, and detection. Recent developments of these methods now enable researchers to uncover protein mechanisms in vivo that may previously have only been hypothesized. These methods are also useful for discovering new biology because how proteins regulate systemic interactions is not well understood in most cases, such as how they travel through the bloodstream to distal targets or cross the blood–brain barrier. Genetic sequencing of DNA and RNA have enabled many great discoveries in the past 20 years, and now, the protein methods described here are creating a more complete picture of how cells to whole organisms function. It is likely that these developments will generate another transformation in biomedical research and our understanding of the brain and will ultimately allow for patient-specific medicine on a protein level.

## Background

Proteins play the most active roles in cellular function, yet technologies to study molecules at the DNA and RNA level have developed at a much faster pace compared to current methods available for studying proteins [1]. This limitation has led to slow understanding of cellular function in the contexts of homeostasis, disease, and dysfunction. Over the past decade, antibody/aptamer detection methods and mass spectrometry have emerged as the primary tools driving the detection of proteins on a cellular and tissue level [2]. As emerging biological questions increasingly require an understanding of intercellular, inter-organ, and systemic interactions, the need to accurately track the movement of proteins and understand their effects across tissues and organs becomes essential. Blood plasma contains proteins secreted from all tissues, even those that are difficult to access, such as the brain, and thus provides a rich source of biomarkers useful for determining patient health and potentially even organ-specific information. For example, a blood test for tau combined with two short cognitive tests and genotyping for Alzheimer’s disease can now predict mild cognitive impairment with approximately 90% accuracy [3].

Protein tracking is useful for understanding how proteins secreted into the bloodstream can affect other tissues exposed to them through the vasculature [4]. The brain appears to be meaningfully affected by proteins secreted from peripheral tissues into the bloodstream, despite the blood–brain barrier (BBB) traditionally being thought of as highly impermeable to proteins or even small peptides [5]. For example by using radiotracers, neurotrophins such as BDNF and NGF were shown to cross the BBB, likely due to specific transport systems for these proteins [6, 7]. During aging and neurodegenerative disease, the BBB exhibits altered transport properties facilitating increased non-specific uptake into the brain of proteins including IgG, fibrinogen, and thrombin [5, 8]. The ability to detect protein transport into different brain regions under various pathological conditions is crucial for understanding the influence of plasma proteins on brain health and neurodegeneration.

Another example of the need for protein tracking is intercellular communication between the same or different cell types within a single tissue. Proteins mediate intricate signaling pathways between cells to coordinate orchestrated responses, such as with immune cell infiltration or responses to bacterial/viral infection [9]. The BBB itself is composed of endothelial cells, astrocytes, and smooth muscle cells that each secrete proteins that are critical for communication and maintenance of this layer [10–13]. In disease contexts, proteins may become mislocalized or dysregulated, affecting cell signaling and other important biological processes [14]. Thus, tracking proteins is useful for evaluating the state of a cell or cell system and may uncover novel opportunities for therapeutic development.

Recently, more tools have been developed to label and track specific proteins or proteomes for disease and BBB model systems. These methods are still under development and often require overcoming technical hurdles to be used. Therefore, we will describe here how these tools can be practically used to explore important aspects of proteins in biology, offering an overview of capabilities with current and upcoming technologies. We will mainly discuss the use of these methods in the context of BBB transport as a particularly interesting barrier to protein movement, but these tools may be applied in many other biological contexts, often even more easily. Some of these tools may be used in humans either now or in the future to diagnose disease or directly understand human biology, but perturbing entire proteomes may have unforeseen consequences that necessitate tools for non-human model systems. Ideally, tools used in humans should be transient and reversible, such as non-covalent binders of proteins that are rapidly cleared from the body in hours to days. Proteins can also be sampled with or without labeling from interstitial fluid microdialysis or directly from other fluids (e.g. blood, cerebrospinal fluid, urine, saliva). In-human protein labeling and tracking tools include isotopes, binders, nanoparticles, and tracers that associate with or mimic proteins of interest, but these options almost always do not permanently change proteins or proteomes as some model system tools do. We will first describe useful methods of protein labeling, then elaborate on how labeled and unlabeled proteins can be tracked and detected in model systems. We cite specific examples as references to refer to that should allow researchers to quickly adapt a tool of interest to their research with both methods and details. These methods are useful for studying protein function in biological contexts, a critical part of the ultimate goal in understanding the complexity of model organisms and human health that is still elusive to understanding.

## Protein labeling

### Tracers

Protein labeling has a long history with some tools still remaining relevant today. Evans blue (EB) described 100 years ago in 1920 is an example of a dye that is still widely used, in fact even more frequently over the past decade, as it is an inexpensive and clear way to visualize the permeability of organs, critical to understanding the BBB [15, 16]. EB functions by binding albumin, the most abundant protein found in blood plasma. Under normal physiological conditions in young mice, albumin effectively does not cross the BBB. However, damage to the BBB increases the permeability of blood proteins into the brain, which can be visualized by increased EB staining in brain tissue during ischemic stroke, for example [17]. Leakage of intravenously injected EB into the brain was also reported during normal aging in 12 to 24 month old mice, but not 2 to 4 month old mice, suggesting EB is also a useful tool even under more subtle contexts, although care should be taken as old brains often exhibit autofluorescence that may confound the observation of fluorescence from EB [8, 18]. In addition, lung endothelial cells can be used as positive controls for tracers or proteins in BBB experiments as they do not possess the restrictive barrier seen in the brain [19, 20]. Other tracers are available to also track leakage through the BBB, including fluorescein, trypan blue, biotin, horseradish peroxidase, IgG, and isolectin [15, 19]. Labeled dextrans are a tracer of particular interest as the size (10 to 70 kDa), wavelength, and brightness can be easily controlled to examine how different molecular weight molecules interact with the BBB [19, 20].

MRI contrast agents also were originally used in free circulation, especially Gd^3+^ [21, 22]. Like EB, Gd^3+^ demonstrates some weak protein binding, often for proteins that naturally coordinate other ions [23]. To avoid accumulation in the body, chelators such as DTPA and DOTA are now often used to facilitate contrast agent removal [24]. These chelators can also be used to directly chemically attach Gd^3+^ or other lanthanides to a protein of interest to track specific proteins, rather than being a simple free circulating contrast agent [25, 26]. Positron emission tomography (PET) using radionuclides, including ^64^Cu, ^68^ Ga, and ^177^Lu, chelated by DOTA can similarly be used to directly visualize proteins and cells *in vivo* [27, 28].

### Genetically encoded labels

**Fig. 1:**
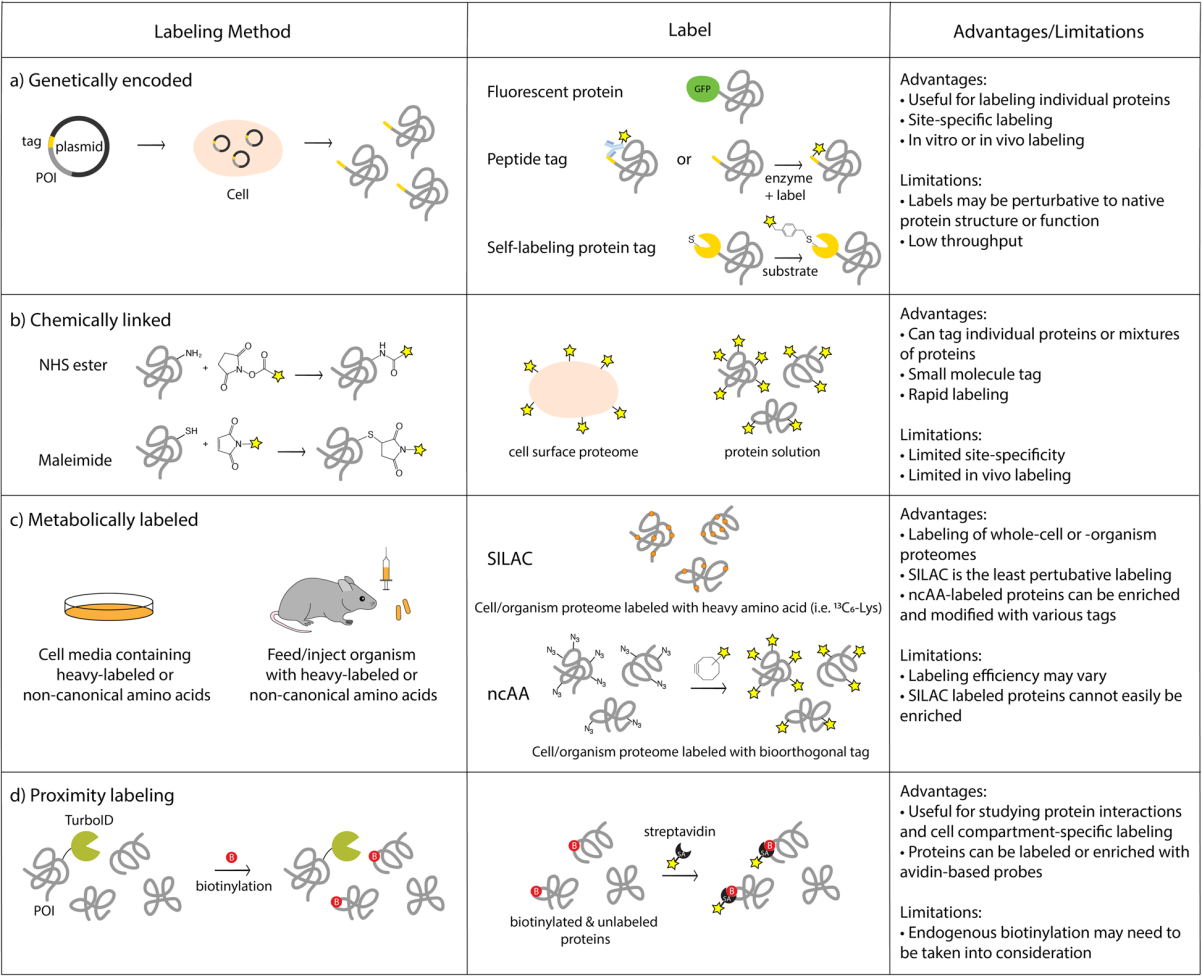
Tools for protein labeling. Summary of important protein labeling methods discussed in the review and their key advantages and limitations are highlighted here

Tools to label proteins have been long used to track individual proteins, of which many are genetically encoded (Fig. 1A). Most commonly, GFP and other fluorescent proteins can be co-expressed attached to a protein of interest as a fusion protein [29]. Although one of the most simple methods of protein labeling, this approach still has advanced uses, such as in super-resolution and single-molecule live-cell microscopy [30, 31]. Intracellular localization of proteins can often be observed via this approach, as well as changes in localization upon various stimuli. In terms of obtaining physiologically accurate results, the major drawback to these fusion proteins is the large size of the fluorescent protein label which can often interfere with biological functions, such as secretion, enzymatic activity, or interactions [32].

Alternative labels to GFP fusion proteins include epitope tags such as FLAG, hemagglutinin (HA), and Myc tags [33, 34]. These tags are only a few amino acids, greatly reducing the probability of interfering with protein function. The disadvantage here is that exogenous antibodies must be added for detection, increasing the complexity of the experiment. However, highly specific antibodies for these tags are readily available, minimizing off-target labeling for proteins where antibodies are untested or unavailable and enabling fast and accurate experimental results.

Some peptide tags allow enzymatic modifications to target proteins, such as methods using formylglycine-generating enzyme (FGE), sortase, biotin ligase, and lipoic acid ligase [35]. In these methods, a short peptide sequence is introduced into a specific site in the protein of interest and subsequently acted upon by the enzyme and any additional substrates to generate a new chemical group that can be selectively labeled. For example, FGE recognizes a 6–13 residue peptide tag and converts a cysteine within the motif into an aldehyde group that can be further tagged with probes using hydrazide and aminooxy chemistry [36]. These chemoenzymatic methods are useful for site-specifically installing a number of different functionalities onto proteins but have limited uses in vivo.

Other genetically encoded tags such as glutathione-S-transferase (GST), chitin binding protein (CBP), maltose binding protein (MBP), and His tags should generally be avoided for protein tracking, although they have an advantage in affinity protein purification [33, 34]. GST, CBP, and MBP are all proteins that may interfere with protein function and distribution like GFP, but without the advantage of fluorescence. Also, His tags are small like FLAG, HA, and Myc tags, but, in our experience, antibodies may not always be as effective in specific His tag identification.

SNAP-tags, CLIP-tags, and HaloTags are self-labeling protein tags that provide another alternative to genetically encoded fluorescent proteins [37, 38]. These tags allow for the attachment of a small molecule fluorophore, biotin, or bead to a protein of interest by covalently linking these molecules with the chemical group that reacts with the genetically encoded tag. This application can be useful in specialty applications for some techniques, such as a correlative light and electron microscopy [39]. Of note is that HaloTags have been reported to have ninefold higher signal than SNAP-tags, which can be important for super-resolution microscopy [40].

The major advantage of genetically encoded tags is the high degree of control over the location of the tag within the protein. Small peptide tags have allowed these labels to be applied with minimal interference to protein activity or distribution and to be functionalized with diverse molecules useful in many contexts. While these tools have been successfully used for site-specific labeling of individual proteins, they cannot easily be applied for efficient labeling of many proteins at a time and often face limitations for use in vivo.

### Chemically linked labels

Chemical labeling of proteins is a useful way to label specific proteins or even entire proteomes (Fig. 1B). Chemical labeling allows for smaller, potentially less disruptive tags for downstream analysis in experiments [5, 41]. Through chemical labeling, the protein or proteome can be enriched and/or identified at a later time and, in this way, tracked from its original source.

Two of the most widely used chemical bioconjugation methods for proteins are N-hydroxysuccinimidyl (NHS) esters and maleimides [42]. NHS esters label primary amines, targeting lysine side chains and the N-termini of proteins and peptides. However, care should be taken as other residues such as tyrosine and serine can be labeled if reactions occur for too long or at too high concentrations [43]. Maleimides label the side chain of cysteines, but oxidation of cysteines into disulfides can block the reaction [44]. Both reactions can be used independently to confirm that the chemical modification does not interfere with protein function. In our experience, NHS esters are easier to use at first as the reaction is more reliable, especially since lysines often occur on the surface of proteins and cysteines are more likely to be inaccessible [5]. Maleimides can then be used to confirm findings. Maleimides can also be used for site-specific labeling of proteins by first mutating all cysteine residues to serine, which is chemically similar, and then creating cysteine point mutations throughout a specific protein of interest [45]. In addition to lysine and cysteine modifications, many chemical reactions have been developed to specifically label other residues including methionine, tyrosine, aspartate, glutamate, and the N- or C- terminus. Overall, these techniques have been tuned to have increased control on site selectivity but are still limited in terms of specificity of labeling compared to genetically encoded tags.

Critical for visual and spectroscopic tracking of proteins in many applications is the selection of small molecule fluorophores. Small molecule fluorophores may interact with proteins, lipids, and other biomolecules through hydrophobic and electrostatic interactions, leading to inaccurate measurement of fluorescent signals [46]. The brightness of the molecule, protein concentrations, and wavelength, such as autofluorescence of tissues at the GFP/488 nm wavelength, are also important considerations. In our experience, bright Atto dyes in the red shifted region generally give the best results, but controls using other dyes or other proteins should be included to minimize false positives. In addition to fluorophores, many other chemical tags can be linked to proteins using the methods described above, including crosslinkers and enrichment tags. Chemical labeling is very effective for labeling cell surface proteomes and fluid solutions such as blood plasma or CSF that can be labeled ex vivo and re-injected in vivo if needed. Our research group has applied these methods to label the mouse plasma proteome and assess changes in BBB transport during aging *in vivo* [5].

### In vivo SILAC and noncanonical amino acid labels

Methods that minimize changes to proteins are typically less perturbative to protein localization and function. The most non-perturbative labeling method is nonradioactive, isotopic labeling, such as SILAC (Fig. 1C). This method enables whole proteome labeling via the incorporation of amino acids containing heavy isotopes such as ^13^C, ^15^ N, and ^2^H [47]. Heavy-labeling of proteins can then be detected using mass spectrometry based on a defined mass shift compared to the naturally occurring lighter isotopes in proteins. While SILAC is easily carried out in cell culture by growing cells in media containing heavy amino acids, labeling in vivo in mice or other mammals is more difficult due to the expense required to provide several weeks or months of chow for labeling and the inability to easily remove natural isotopes. However, ^13^C_6_-Lysine feed has been shown to provide sufficient labeling in mice after about 2 weeks, at a cost for feed of ~ $700 per mouse, which may be a reasonable cost for some experiments [48]. While SILAC is extremely useful for distinguishing proteins from different sources for mass spectrometry applications, there are two main disadvantages of this technology. Firstly, SILAC-labeled proteins cannot be functionalized easily for other applications such as histology. Secondly, and perhaps more importantly, SILAC labeled proteins cannot be enriched, so isotopically-labeled proteins at very low concentrations cannot be easily detected, even with mass spectrometry.

A new alternative to SILAC mice is the use of noncanonical amino acids (ncAAs) which can be incorporated into proteins using native or engineered protein translation machinery (Fig. 1C). ncAAs are often used to introduce bioorthogonal tags that can be labeled or enriched using click chemistry. Azidohomoalanine (AHA) and homopropargylglycine (HPG) are methionine analogs that can be incorporated into wild type mouse or human cells and tissues by endogenous methionyl-tRNA synthetases, with minimal changes to protein biology [49, 50]. AHA or HPG can be provided as feed, in drinking water, or injected, but we have noticed toxicity with intraperitoneal injections in mice, especially at concentrations at the maximum levels of what was reported previously [50]. These ncAAs are best used when provided in mouse feed containing 0% methionine to reduce methionine incorporation [49]. We have successfully used both AHA and HPG to label tissues and blood plasma, and, in our experience, AHA labeling is noticeably stronger and less toxic than HPG, so AHA is greatly preferred. One disadvantage of AHA/HPG mouse labeling is that specific cell types or tissues cannot be targeted as these ncAAs use the native mouse methionyl-tRNA synthetase for incorporation, so labeling occurs throughout the entire mouse. We also observe moderately less labeling in the brain, likely due to the BBB occluding the ncAAs.

To overcome the challenge of tissue-specific or cell-specific incorporation of ncAAs in animals, the use of the pyrrolysyl-tRNA synthetase/tRNA pair (PylRS/tRNA^Pyl^) that targets the amber codon was first implemented, as only cells or tissues expressing this orthogonal pair will be labeled with the corresponding ncAA [51]. Since PylRS/tRNA^Pyl^ does not occur in mammals, it can incorporate novel ncAAs into cells or tissues only where it is expressed. PylRS/tRNA^Pyl^ has mostly been used to introduce ncAAs site-specifically into proteins genetically modified to contain the amber codon in *E. coli* until recently, but this system has an advantage of now > 150 ncAAs engineered for use through PylRS mutations [51, 52]. The lab of Jason Chin has expanded the PylRS/tRNA^Pyl^ pair into mice and other eukaryotes, with their SORT-M system. This technology can also target any amino acid codon, rather than only the amber codon, by mutating the tRNA^Pyl^ anticodon to correspond to the targeted codon. This system has also been further improved by mutations that increase the tRNA^Pyl^ stability in mammalian cells [52]. Two versions of ncAAs, one containing a methylcyclopropene side chain and one containing a terminal alkyne can be used with the SORT-M system to facilitate bioorthogonal click chemistry with desired probes. The SORT-M system is the most diverse system for use in eukaryotes, although in our hands we have found it to be less robust for strong labeling as the tRNA stability is still limited in vivo. However, this system has now been clearly demonstrated to be a powerful tool in mice with recent method developments of the Chin lab [53].

To overcome the limitation of tRNA^Pyl^ stability or expression, certain aminoacyl-tRNA synthetase (aaRS) mutants exist that can incorporate ncAAs into mammalian cells without requiring the expression an exogenous tRNA. The lab of David Tirrell found a mutant mouse methionyl-tRNA synthetase (MmMetRS_L274G_), allowing the incorporation of the azide-containing azidonorleucine (Anl) at methionine sites in mice [54, 55]. We have also recently discovered a mutant yeast tyrosyl (ScTyrRS_Y43G_) and mouse phenylalanyl (MmPheRS_T413G_) tRNA synthetase that can incorporate 3-azidotyrosine at tyrosine sites and 4-azidophenylalanine at phenylalanine sites, respectively, in mice [56, 57]. The MmMetRS_L274G_, ScTyr_Y43G_, and MmPhe_T413G_ all are efficient at amino acid incorporation in our hands. However, we have observed the MetRS and TyrRS mutants seem to have the most robust incorporations of their respective ncAAs in mice compared to PheRS or the SORT-M system. The TyrRS also has the advantage of a short gene sequence, which is important for the limited packing capacity of adeno-associated viruses (AAVs) for viral genetic transduction. The TyrRS also allows ncAA incorporation at near maximum efficiency even at low concentrations (15 µM of 3-azidotyrosine) [56]. Overall, SORT-M, MmMetRS_L274G_, ScTyr_Y43G_, and MmPhe_T413G_ all have shown great promise for cell and tissue-specific proteome labeling. Using a multiple of these four approaches can also be useful to increase proteome coverage and to ensure reproducibility of results with different ncAAs incorporated into different amino acid sites.

The lab of Alice Ting has also produced other complementary methods using proximity labeling with BioID or TurboID, which both utilize a biotin ligase linked to a protein of interest or cellular localization sequence to attach biotin to nearby surrounding proteins (Fig. 1D) [58–60]. As pulling down biotinylated proteins using streptavidin is a widely-used, low background, and high-specificity technique, it can offer easy adoption and reliable results. One caveat is that endogenous biotin is consumed by the method, so care must be taken especially when used in vertebrates. There are also endogenously biotinylated proteins, but these can be subtracted out as they are also present in control samples. This approach has been used in vivo to successfully label tissue-specific secretomes in mice, which can then be detected in serum [60]. This approach also allows for a powerful proximity labeling technique, in which the biotin ligase is attached to a single protein to identify the nearby interaction partners, even revealing many interaction partners that are unknown [61].

## Protein tracking

### Single protein tracking

As discussed above, there are many methods to label proteins (Fig. 1). These labels can then be used to track proteins from their source of production to another location. To do this in vivo, labeling at a specific cell or tissue type and identifying the labeled protein at another location is required. To achieve this goal in mice, the Cre-loxP system can be harnessed to drive genetic changes allowing labeling only in a certain cell type [62]. The aaRS mutants described above can be introduced into a transgenic mouse where expression is normally repressed by a stop cassette floxed by loxP sites. Cells expressing Cre will remove the stop cassette to allow expression of the aaRS and the incorporation of the corresponding ncAA only in those specific cells. For example, Cre can be expressed specifically in brain endothelial cells [63], possibly allowing labeled proteins made in these cells to be tracked to either the abluminal brain side or the luminal blood side.

Furthermore, GFP and similar genetically encoded fluorophores have long been a useful tool for tracking in several contexts. When inserted into the locus of growth hormone in transgenic mice, GFP can be used to visualize secretion in the pituitary [64]. GFP can be combined with the Cre-loxP system to easily visualize the desired protein in a particular cell type. Mice where all cells have been labeled with GFP have also been used in the context of parabiosis to track immune cells from the labeled mouse to the unlabeled mouse [65, 66].

An alternative to the Cre-loxP system is the use of viruses harboring tissue or cell type-specific promoters to deliver gene constructs, like a bioorthogonal aaRS or tagged protein, into specific cell types. Lentivirus and adeno-associated virus (AAV) are the most commonly used viruses for gene delivery into cultured cells or in vivo. Lentivirus is a retrovirus that integrates its genomic information into the host’s genome which is advantageous for making stable cell lines in vitro but can cause insertional mutations in vivo [67]. AAVs, on the other hand, exhibit non-integrating gene expression and are considered to be safer and are now more commonly used for in vivo and therapeutic gene delivery [68, 69]. Different AAV serotypes have particular tropisms that are important to consider for targeting the infection to certain tissue types. To further control transduction, the gene can be under the control of a cell-specific promoter.

### Proteome tracking

Labeling of proteins using ncAAs has been recently used to track even entire proteomes in vivo. For example, the proteome of excitatory neurons was specifically labeled in mice using MmMetRS_L274G_ under the control of the CaMK2a promoter [70]. These mice exhibited a distinct protein expression pattern when placed in an enriched environment compared to a standard cage [70]. A separate study specifically labeled proteins in hippocampal neurons in mice trained for active place avoidance via foot shocks in a quadrant of a circle to induce long-term memory (LTM) formation. Known memory proteins were found to be expressed during LTM formation, as well as new mRNA splicing-associated proteins not previously implicated in LTM [71]. In another study, bioorthogonal labeling of a mouse expressing MmMetRS_L274G_ parabiosed with an unlabeled mouse was used to show infiltration of labeled blood protein factors into the muscle of the unlabeled mouse [55]. This study shows that protein signaling through the blood stream can affect distal tissues, which can be tracked with ncAAs. Likewise, tracking the labeled melanoma proteome in mice using ScTyr_Y43G_ or MmPhe_T413G_ can show the impact of cancer-related proteins on distal tissues, an example of how protein tracking can determine the impact of disease on indirectly affected tissues [56].

One advantage of ncAAs over genetically encoded fluorophores like GFP, is the ability to use more diverse imaging techniques to track proteins. GFP labeled proteins have long been imaged in live cells, a simple and reliable method of tracking [72, 73]. Super resolution techniques where brighter, synthetic fluorophores are preferred, are facilitated by bioorthogonally tagged ncAAs that can be reacted with fluorophores via click chemistry [74, 75]. Click tags have also been used to track proteins in vivo, such as for localization of proteins within a mouse through fluorescent, infrared, or other imaging [5, 76, 77].

## Protein detection

In addition to the above imaging methods, proteins can be detected using histology methods. Proteins have been traditionally imaged using histology by either antibodies against the protein of interest or a tag attached to the protein of interest. Click chemistry with ncAAs is a new, important way to detect proteins using histology. Like protein tags and chemical linked labels, ncAAs can be used to detect proteins where there is no effective antibody for a certain protein because a fluorophore can be attached to any targeted protein that has the required ncAAs incorporated [78]. In addition, ncAAs allow nascently made proteins from specific cells to be detected via pulse chase experiments, allowing both for differentiation of newly synthesized proteins from older proteins, relevant to disease, such as in protein aggregates, and for targeted applications where a certain cell type is being studied in complex tissues, such as the brain [54].

Mass spectrometry is becoming an increasingly more powerful method to detect proteins and proteomes. SILAC for differential detection of proteins by mass spectrometry is one such application that has been widely used to make biological discoveries [48]. However, SILAC does not allow for cell-specific labeling or enrichment of proteins of interest, which can limit detection of proteins by more than two orders of magnitude [79]. By using ncAAs, enrichment is possible when labeling full proteomes [56]. ncAAs only modify natural amino acids by a few atoms, allowing for minimal changes in protein function and overall physiology, similar to SILAC and unlike larger protein tags. Practically, we have found the best way to enrich proteins ex vivo is by using clickable biotin with a cleavable linker [80]. In our experience, this method enriched the largest number of labeled proteins or peptides, while minimizing background by offering the ability to filter out unlabeled peptides computationally after mass spectrometry results are obtained. We have found the software packages MaxQuant and Perseus useful because they contain the ability to create these filters [5, 81, 82]. Mass spectrometry has gone from femtomolar to attomolar detection in recent years, enabling detection of even low concentrations of protein [83, 84]. As the concentrations of proteins in brain fluid and tissue can be even lower, especially after crossing from blood to the brain, enrichment allows for an even lower range of detection.

Autoradiography is another useful method for protein detection. In this technique, proteins can be labeled with radioisotopes and injected into animals. One common method is to chemically conjugate the desired proteins to the chemical chelator DOTA which can further react with radionuclides including ^64^Cu, ^68^ Ga, and ^177^Lu. Tissues can then be analyzed ex vivo to determine biodistribution of proteins or even cells. This technique has successfully been used to detect the transport of target proteins and plasma into the brain or spinal cord after crossing the BBB [27, 65].

In line with mass spectrometry proteomics that can detect thousands of proteins, antibody and aptamer arrays have been also used for multiprotein detection. The main advantage over mass spectrometry here is that arrays can have high sensitivity to proteins even at low concentrations and have a lower technical barrier to usage. However, arrays have fallen behind in many respects to mass spectrometry because arrays are only as good as the antibodies or aptamers they contain. If many antibodies/aptamers are not specific to the desired protein, false positives or false negatives can often appear. New protein detection platforms have addressed these issues by developing probes with very high sensitivity and specificity for protein targets, such as platforms multiplexing thousands of proteins by using pairs of antibodies combined with rolling circle amplification PCR employed by OLINK or aptamer arrays developed by Somalogic [85, 86]. Data from these platforms can be processed in analyzed with packages in R [87].

New methods are upcoming that will advance protein detection even further. Notably, highly parallel identification of single protein molecules offers the promise of RNAseq level sensitivity for detection of proteins [88]. This method would theoretically outperform even newer mass spectrometry methods, although it is still in early development. Detection will also be improved by methods allowing for alternative enrichment strategies outside of click chemistry, such as with biotinylation of proteins directly in live animals via TurboID [59]. Spatial omics may also be combined with these methods in the future to determine if proteins are detected in cells without the corresponding RNA, likely indicating the protein originated from another cell [89]. Data from all these tools increasingly offer ample opportunities for machine learning and multi-modal data analysis, as almost all individual molecules in all cells, fluids, and tissues are beginning to be accessible. With newer technologies, the ease of labeling, tracking, and detecting proteins will allow for proteomics discoveries on the scale of, and perhaps combined with, RNAseq.

## Conclusion

RNAseq has been a major force driving our understanding of homeostasis, aging, and disease states of cells and tissues. However, protein changes often do not correlate well with RNAseq and require the use of protein tracking and detection methods for understanding of the biology underlying these processes in organisms. Tools to measure and detect proteins have developed significantly over the past decade, but they have not yet reached their prime to understand the full span of the proteome. Newer tools for protein detection described here offer temporal and cell-specific proteome coverage across a large range of up to thousands of proteins. Newer protein labeling methods offer more precise control over the proteome, even in vivo in mice. Mass spectrometry developments have enabled these technologies even more by increasing proteome coverage. By applying these tools to model systems, new mechanisms of mammalian biology that have implications in the detection and treatment of human disease will be uncovered. These tools will also likely be applied directly to humans where effects are transient or produce no discernable negative effects in model systems. We predict that now as protein labeling and detection methods are becoming accessible to researchers, our understanding of the proteome, including the plasma proteome, will once again transform our insight into the biology of living organisms and diseases in the coming years.

## Data Availability

Not applicable.

## Abbreviations

aaRS: Aminoacyl-tRNA synthetase
AAV: Adeno-associated virus
AHA: Azidohomoalanine
Anl: Azidonorleucine
BBB: Blood–brain barrier
CBP: Chitin binding protein
EB: Evans Blue
FGE: Formylglycine-generating enzyme
GST: Glutathione-S-transferase
HA: Hemagglutinin
HPG: Homopropargylglycine
LTM: Long-term memory
MBP: Maltose binding protein
MmMetRS_L274G_: Mutant mouse methionyl-tRNA synthetase
MmPheRS_T413G_: Mutant mouse phenylalanyl tRNA synthetase
ncAA: Noncanonical amino acids
NHS: N-hydroxysuccinimidyl
PET: Positron emission tomography
PylRS/tRNA.^Pyl^: Pyrrolysyl-tRNA synthetase/tRNA pair
ScTyrRS_Y43G_: Mutant yeast tyrosyl tRNA synthetase
SILAC: Stable-isotope labelling by amino acids in cell culture
RNAseq: RNA sequencing
